# The laval questionnaire: a new instrument to measure quality of life in morbid obesity

**DOI:** 10.1186/1477-7525-9-66

**Published:** 2011-08-15

**Authors:** Fanny Therrien, Picard Marceau, Nathalie Turgeon, Simon Biron, Denis Richard, Yves Lacasse

**Affiliations:** 1 Centre de recherche, Institut universitaire de cardiologie et de pneumologie de Québec affilié à l'Université Laval, 2725 Chemin Ste-Foy, Québec, Québec, G1V 4G5, CANADA

## Abstract

**Background:**

Our recent review of the literature uncovered eleven obesity-specific quality of life questionnaires, all with incomplete demonstration of their measurement properties. Our objective was to validate a new self-administered questionnaire specific to morbid obesity to be used in clinical trials. The study was carried out at the bariatric surgery clinic of Laval Hospital, Quebec City, Canada.

**Methods:**

This study followed our description of health-related quality of life in morbid obesity from which we constructed the Laval Questionnaire. Its construct validity and responsiveness were tested by comparing the baseline and changes at 1-year follow-up in 6 domain scores (symptoms, activity/mobility, personal hygiene/clothing, emotions, social interactions, sexual life) with those of questionnaires measuring related constructs (SF-36, Impact of Weight on Quality of Life-Lite, Rosenberg Self-Esteem Scale and Beck Depression Inventory-II).

**Results:**

112 patients (67 who got bariatric surgery, 45 who remained on the waiting list during the study period) participated in this study. The analysis of the discriminative function of the questionnaire showed moderate-to-high correlations between the scores in each domain of our instrument and the corresponding questionnaires. The analysis of its evaluative function showed (1) significant differences in score changes between patients with bariatric surgery and those without, and (2) moderate-to-high correlations between the changes in scores in the new instrument and the changes in the corresponding questionnaires. Most of these correlations met the *a priori *predictions we had made regarding their direction and magnitude.

**Conclusion:**

The Laval Questionnaire is a valid measure of health-related quality of life in patients with morbid obesity and is responsive to treatment-induced changes.

## Background

Obesity is defined by a body mass index (BMI) greater than 30 kg/m^2^; when the BMI is greater than 40 kg/m^2^, obesity is morbid [1]. Morbid obesity is associated with the onset or the deterioration of several physical health problems, including cardiovascular diseases, type II diabetes, dyslipidemia, sleep apnea, respiratory failure, osteoarthritis, infertility, and cancers of several organs including colon, breast, prostate and endometrium [1,2]. Also, morbid obesity is often complicated by depression and impaired quality of life [1,2]. In addition, the risk of death of obese individuals is increased by 50 to 100% compared with normal-weight individuals [3].

Treatment strategies for obesity include diet, physical activity, behavioural therapy, pharmacotherapy and surgery [1]. In the evaluation of these strategies, more emphasis has been given to weight loss, co-morbidities and mortality than to disease-specific quality of life [4]. Although a relationship between BMI and quality of life impairment has been noted [4,5], this association was weak and depended on a variety of factors including gender, race, treatment-seeking status, treatment modality and BMI, the latter explaining only about one fourth of the variance [6,7]. Therefore, BMI or the magnitude of weight loss after a given intervention do not necessarily represent appropriate surrogate outcomes to quality of life that needs to be measured directly.

Although generic instruments for measuring health-related quality of life such as the Medical Outcome Survey - Short Form 36 (MOS-SF-36) [8] provide useful information, they are not designed to measure the specific range of health-related problems experienced by individuals with morbid obesity. A recent study by Kolotkin et al. [9] found differences between weight-related and generic measures of health-related quality of life in a one-year weight loss trial, emphasizing the potential value of using more than one measure in a trial, including a disease-specific questionnaire. Our review of the literature uncovered eleven obesity-specific quality of life questionnaires, all with incomplete demonstration of their respective measurement properties [10]. Only three targeted morbid obesity [11-13]. Construct validity was properly studied in three questionnaires [14-16]. Demonstration of responsiveness from independent randomized controlled trials was available for two of the eleven questionnaires [17,18]. The interpretability of the eleven questionnaires was limited.

We previously described the impact of morbid obesity on the quality of life of patients seeking surgical therapy [19]. This study identified the domains of quality of life most frequently affected by morbid obesity from which we constructed the Laval Questionnaire, a new self-administered questionnaire specific to morbid obesity. The objective of this study was to examine the validity, reliability, responsiveness and interpretability of this new questionnaire to be used in clinical trials.

## Methods

### The Laval Questionnaire

The Laval Questionnaire is a 44-item questionnaire that is meant to be used as an evaluative instrument - that is, as a clinical outcome in clinical trials. The Laval Questionnaire was developed in French. The methodology used for the construction of the questionnaire was described elsewhere [19]. The items having the most important impact on quality of life clustered into 6 domains: (1) symptoms, 10 items; (2) activity/mobility, 9 items; (3) personal hygiene/clothing, 5 items; (4) emotions, 11 items; (5) social interactions, 7 items and (6) sexual life, 2 items. Each domain is scored on a 7-point Likert scale, higher scores meaning better quality of life. The patients are asked to indicate how their obesity affected their life over the last four weeks. Its administration takes on average 10 minutes.

### Study population

This validation study also took place in French in Laval Hospital (Institut universitaire de cardiologie et de pneumologie de Québec, Canada), the busiest Canadian bariatric surgery center with 500 interventions performed yearly. Patients were selected for surgery in strict accordance with the National Institutes of Health guidelines [1]. From September 2007, two groups of consecutive adult patients with morbid obesity awaiting bariatric surgery were included. The "treatment group" consisted of patients for whom the surgery was planned within the next 8 weeks. The surgery consisted in a biliopancreatic diversion with duodenal switch [20]. The "control group" included patients waiting for surgery but not to be operated on within a year. There was no exclusion, i.e., no limit of age or BMI was imposed and patients with co-morbidities (such as obstructive sleep apnea, diabetes or osteoarthritis) were also included. This study received approval from the Ethics Committee of our institution.

### Validation study

Initially, all patients completed the Laval Questionnaire at study entry (Time 1) and, at the same time, the French version of 4 other questionnaires measuring constructs related to those measured by the Laval Questionnaire:

*• MOS-SF-36 *[8]: The MOS-SF-36 is a generic self-completed questionnaire that measures 8 dimensions of health: physical functioning, role limitation due to physical problems, role limitation due to emotional problems, social functioning, mental health, energy/vitality, bodily pain and general health perceptions.

*• Impact of Weight on Quality of Life-Lite (IWQOL-Lite) *[21]: The IWQOL-Lite is a 31-item evaluative self-completed questionnaire specific to obesity that measures 5 domains of quality of life: physical function, self-esteem, sexual life, public distress and work.

*• Beck Depression Inventory (BDI) *[22]: The BDI is a 21-item traditional instrument that was developed specifically to identify depression. It has been extensively used as an evaluative instrument to monitor response to therapy in clinical trials.

*• Rosenberg Self-Esteem Scale (SES) *[23]: The SES is a 10-item self-report measure of global self-esteem. It consists of 10 statements related to overall feelings of self-worth or self-acceptance.

Two weeks later, assuming clinical stability over this period of time and before any intervention, we administered again the Laval Questionnaire to all patients in order to examine its test-retest reliability (Time 2). The whole set of questionnaires was again completed 1 year (± 1 month) after surgery for the treatment group, and one year after the initial evaluation for those still on the waiting list (time 3). All questionnaires were self-administered. At time 2 and time 3, the respondents remained unaware of their previous responses.

### Statistics

#### Baseline characteristics, questionnaires scoring and sample size

Descriptive statistics (proportions, means and standard deviations) were used to describe the study population at baseline. Chi-square and t-tests were used to compare the baseline characteristics of the "treatment" and "control" groups when appropriate. Individual items of the Laval Questionnaire were equally weighted. The results were expressed as the mean score per item (ranging from 1 to 7) within each domain. The other questionnaires were analyzed as advocated by their respective authors. We computed that at least 45 patients were needed if moderate (r = 0.50) but statistically significant correlations were to be detected in the baseline discriminative analyses at the 0.01 level (β error: 0.15) [24].

#### Reliability and internal consistency

"Test-retest reliability" was determined by correlating the results obtained at Time 1 and Time 2 using intraclass correlation coefficients. Internal consistency (the extent to which different items in an instrument are measuring the same construct) was determined for each domain using Cronbach's alpha statistics [25].

#### Discriminative properties

In this analysis, we examined the extent to which the Laval Questionnaire can distinguish among groups of patients. Cross-sectional construct validity was evaluated by correlating baseline scores with other related measures, and by showing that these correlations conformed with what one would expect if the questionnaire was measuring what it was supposed to measure. Throughout the regression analyses, given the multitude of comparisons involved, statistical significance was set at the 0.01 level.

#### Evaluative properties

In this analysis, we examined the extent to which the Laval Questionnaire can capture changes in quality of life over time (that is the responsiveness of the questionnaires). This was primarily tested as the ability of the questionnaires to detect statistically significant differences in scores in the patients who were treated over the study period (Time 3 - Time 1) using paired t-tests. Also, we computed the standardized response mean that compares the magnitude of change with its standard deviation [26]. The standardized response mean represents an intuitive estimate of the "signal-to-noise ratio" defining responsiveness. Finally, we examined the ability of the questionnaire to distinguish between groups of patients (treated vs. untreated, i.e., "treatment" vs. control groups) in terms of a change in quality of life during the study period (Time 3 - Time 1) using unpaired t-tests. All differences (T3 - T1) were adjusted for baseline scores. Longitudinal construct validity was also demonstrated by correlating within-subjects changes in quality-of-life scores with within-subjects changes in other quality-of-life indices, and by showing that correlations of changes in different measures conformed with what one would expect if the questionnaire is measuring what it is supposed to measure.

#### Interpretability

For an evaluative instrument, a score is interpretable when it tells the reader whether a particular change in score represents a significant clinical improvement or deterioration [27]. In this analysis, we wished to estimate the minimal clinically important difference (MCID) of the new questionnaire. The MCID is defined as the smallest difference in score which patients would perceive as beneficial and would mandate, in the absence of troublesome side effects and excessive cost, a change in patients' management [27]. To do so, we used the regression method described by Schunemann et al. [28]. We built linear regression models in which the dependent variables were the differences in the Laval Questionnaire's domains scores, and the predictor variables were the differences in scores on the corresponding IWQOL-Lite domains. We estimated MCID only from those domains or instruments for which Pearson's correlation coefficients were 0.5 or greater. From the regression equations, we calculated the score on the Laval Questionnaire that corresponded to the MCID of the IWQOL-Lite (7.7 to 12 on a 100-point scale) [29].

### *A priori *predictions

We formulated *a priori *predictions regarding expected correlations between related measures. The magnitude and direction of these correlations should conform with what one would expect if the new instrument is measuring what it is supposed to measure [30]. At baseline, we anticipated moderate-to-high correlations (0.5 ≤ r < 0.7) between scores in each domain of the Laval Questionnaire and the corresponding instruments. Also, we anticipated weak-to-moderate correlations (0.3 ≤ r < 0.5) between changes in scores in the Laval Questionnaire and changes in the other related questionnaires. The finding that the actual correlations meet these *a priori *predictions would strengthen inferences regarding the validity of the new questionnaire.

## Results

### Patients

The demographic and clinical characteristics of the 112 (67 in the treated group and 45 in the control group) patients who participated in the study are summarized in Table 1. Seventy-four patients were available at 1-year follow-up (48 in the treatment group, 26 in the control group). The baseline characteristics of those available vs. those unavailable at follow-up were not statistically different (data not shown).

**Table 1:** Clinical characteristics of the study population

	Treated (n = 67)	Control (n = 45)	*P *value
Gender, male (%)	14, 21%	12, 27%	0.48

Age (years)*	45.0 (10.2)	43.6 (11.6)	0.56

Body mass index (kg/m^2^)*	52.6 (8.5)	54.4 (9.7)	0.34

Co-morbidities (%)			

• Diabetes	33 (49)	15 (33)	0.09

• High blood pressure	38 (57)	24 (53)	0.81

• Sleep apnea	31 (46)	18 (40)	0.53

• Osteoarthritis	33 (49)	21 (47)	0.74

Living with spouse (%)	36 (54)	29 (64)	0.29

Level of education (years)*	11.8 (2.3)	11.6 (2.3)	0.67

Currently working (%)	29 (43)	27 (60)	0.11

* Values are mean (SD)

### Reliability and internal consistency

Test-retest reliability was determined from the whole cohort (i.e., treated and control patients together, n = 90) who completed the questionnaire two weeks apart. Test-retest reliability was excellent, as indicated by the following intraclass correlation coefficients in each domain: symptoms: r = 0.93; activity/mobility: r = 0.90; personal hygiene/clothing: r = 0.85; emotions: r = 0.90; social interactions: r = 0.87; and sexual life: r = 0.84 (all p values < 0.01). Cronbach's alphas were as follows: symptoms (10 items): 0.84; activity/mobility (9 items): 0.93; personal hygiene/clothing (5 items): 0.78; emotions (11 items): 0.90; social interactions (7 items): 0.86; and sexual life (2 items): 0.65, indicating good internal consistency for all domains of the questionnaire.

### Discriminative properties

The observed cross-sectional correlations supporting the discriminative validity of the questionnaires are shown in Table 2. Except for the Rosenberg Self-Esteem Scale, we observed high correlations between the Laval Questionnaire and the other related measures. Our *a priori *predictions were met in most (19/26) of them.

**Table 2:** Correlations* between the LAVAL Questionnaire and related instruments

	*LAVAL Questionnaire domains*					
	**Symptoms**	**Activity/Mobility**	**Personal hygiene/Clothing**	**Emotions**	**Social interactions**	**Sexual life**

**BMI **(kg/m^2^)	-0.14^†^	-0.27^‡^	-0.25^‡^	-0.09^†^	-0.26^§^	-0.04^†^

**SF-36**						

• Physical functioning		**0.67**^‡^	**0.58**^‡^			

• Role - physical		**0.75**^‡^	**0.59**^‡^			

• Bodily pain	**0.70**^‡^					

• General health perception	**0.57**^‡^					

• Energy/vitality	**0.62**^‡^			**0.61**^‡^		

• Social functioning					**0.58**^‡^	

• Role - emotions				**0.53**^‡^		

• Mental health				**0.71**^‡^		

**IWQOL-Lite**						

• Physical function	**0.69**^‡^	**0.85**^‡^	**0.66**^‡^			

• Self-esteem				**0.80**^‡^	**0.77**^‡^	

• Sexual life						**0.61**^‡^

• Public distress					**0.74**^‡^	

**SES**				0.24^‡^		

**BDI**				**-0.77**^‡^		

* Pearson's coefficients of correlation; the coefficients in bold characters are those that met our *a priori *predictions regarding their direction and magnitude (see text).

† Non significant correlation

‡ p ≤ 0.01

§ p < 0.05

Note: The negative coefficients of correlation obtained with the BMI and the BDI are from the higher scores on these measures indicating worse quality of life.

### Evaluative properties

The ability of the Laval Questionnaire, the IWQOL-Lite and the SF-36 to detect changes is summarized in Table 3. Results are presented as within-group differences in the "treatment" group only. The ability to detect change in the "treatment" group was good for all three questionnaires (all paired t tests: p < 0.001). However, the standardized response means were generally higher with the two obesity-specific questionnaires. Also, in examining the ability of the Laval Questionnaire to distinguish between treated and untreated patients, we did not find any difference between the treated and the untreated groups at baseline (data not shown). However, at follow-up, statistically significant differences were observed (Table 4).

**Table 3:** Rating of change in the Laval Questionnaire and the SF-36 after bariatric surgery (n = 48)

Questionnaires	Mean (SD)	SRM*	25%	Median	75%	Range	**p value**^†^
Laval Questionnaire							

• Symptoms	2.1 (1.2)	1.8	1.4	2.2	3.2	-1.6 - 4.3	<0.0001

• Activity/Mobility	3.2 (1.6)	2.0	2.3	3.4	4.3	-0.2 - 5.6	<0.0001

• Personal hygiene/Clothing	3.4 (1.6)	2.1	2.0	3.4	4.8	0.6 - 6.0	<0.0001

• Emotions	2.3 (1.4)	1.6	1.3	2.3	3.2	-0.4 - 5.6	<0.0001

• Social interactions	2.8 (1.6)	1.8	1.5	2.9	3.9	-1.3 - 5.7	<0.0001

• Sexual life	2.5 (1.7)	1.5	1.0	2.5	4.0	-0.5 - 6.0	<0.0001

**SF-36**							

• Physical functioning	48.5 (27.8)	1.7	30.0	55.0	70.0	-25.0 - 90.0	<0.0001

• Role - physical	50.5 (47.1)	1.1	0.0	75.0	100.0	-75.0 - 100.0	<0.0001

• Bodily pain	27.2 (26.4)	1.0	10.0	31.2	42.5	-34.0 - 82.0	<0.0001

• General health perception	33.0 (27.8)	1.2	15.8	33.0	51.1	-37.0 - 85.0	<0.0001

• Energy/vitality	22.4 (22.6)	1.0	10.0	22.5	43.8	-25.0 - 70.0	<0.0001

• Social functioning	30.2 (35.0)	0.9	0.0	25.0	62.5	-37.5 - 100.0	<0.0001

• Role - emotions	31.2 (56.9)	0.5	0.0	0.0	100.0	-100.0 - 100.0	0.0004

• Mental health	12.6 (22.4)	0.6	-4.0	12.0	28.0	-44.0 - 52.0	0.0004

**IWQOL-Lite**							

• Physical Function	53.0 (24.5)	2.2	34.1	56.8	70.4	-9.1 - 100.0	<0.0001

• Self-Esteem	46.2 (29.8)	1.6	21.4	46.4	67.9	-3.6 - 100.0	<0.0001

• Sexual Life	29.6 (36.9)	0.8	0.0	25.0	51.6	-56.2 - 100.0	<0.0001

• Public Distress	49.9 (27.1)	1.8	25.0	55.0	71.25	-10.0 - 95.0	<0.0001

• Work	41.1 (30.0)	1.4	25.0	37.5	62.5	-25.0 - 100.0	<0.0001

* SRM: standardized response mean = magnitude of change/the standard deviation of change [25]. The larger the standardized response mean, the more responsive to change the questionnaire.

† p value attached to the within-group differences in scores in the patients who were treated over the study period (paired t-tests).

**Table 4:** Ability of the Laval Questionnaire to distinguish treated vs. untreated patients*

	A: Rating of change (time 3 - time 1) in the treated group	B: Rating of change (time 3 - time 1) in the untreated group	Treatment effect (A - B)	***P *value**^†^ (A - B)
• Symptoms	2.1 (1.8 to 2.5)	0.3 (0.0 to 0.6)	1.8 (1.3 to 2.3)	<0.0001

• Activity/Mobility	3.2 (2.7 to 3.6)	0.2 (-0.2 to 0.6)	3.0 (2.3 to 3.6)	<0.0001

• Personal hygiene/Clothing	3.4 (2.9 to 3.8)	0.2 (-0.2 to 0.6)	3.2 (2.5 to 3.9)	<0.0001

• Emotions	2.3 (1.9 to 2.8)	0.7 (0.3 to 1.1)	1.7 (1.0 to 2.3)	<0.0001

• Social interactions	2.8 (2.3 to 3.2)	1.3 (0.0 to 2.7)	1.4 (0.3 to 2.6)	0.0132

• Sexual life	2.5 (2.0 to 3.0)	0.7 (0.2 to 1.3)	1.8 (1.0 to 2.6)	<0.0001

* Results are presented as means (95% confidence intervals).

† p value attached to the group differences during the study period (unpaired t-test)

The correlations supporting the longitudinal construct validity of the Laval Questionnaire are shown in Table 5. Overall, except for the SES, there were moderate to high correlations between the changes in the Laval Questionnaire and the related instruments. Our *a priori *predictions were met in most (15/26) of them.

**Table 5:** Correlations* in ratings of change between the LAVAL Questionnaire and related instruments

	*LAVAL Questionnaire domains*					
	**Symptoms**	**Activity/Mobility**	**Personal hygiene/Clothing**	**Emotions**	**Social interactions**	**Sexual life**

**BMI **(kg/m^2^)	-0.07^†^	-0.18^†^	-0.08^†^	-0.15^†^	-0.22^†^	-0.02^†^

**SF-36**						

• Physical functioning		**0.70**^‡^	0.55^‡^			

• Role - physical		**0.61**^‡^	0.54^‡†^			

• Bodily pain	0.57^‡^					

• General health perception	**0.62**^‡^					

• Energy/vitality	**0.50**^‡^			**0.61**^‡^		

• Social functioning					**0.64**^‡^	

• Role - emotions				0.43^‡^		

• Mental health				**0.61**^‡^		

**IWQOL-Lite**						

• Physical function	**0.73**^‡^	**0.87**^‡^	**0.74**^‡^			

• Self-esteem				**0.72**^‡^	**0.60**^‡^	

• Sexual life						**0.53**^‡^

• Public distress					**0.74**^‡^	

**SES**				0.05^†^		

**BDI**				**-0.71**^‡^		

* Pearson's coefficients of correlation; the coefficients in bold characters are those that met our *a priori *predictions regarding their direction and magnitude (see text).

† Non significant correlation

‡ p < 0.01

Note: The negative coefficients of correlation obtained with the BMI and the BDI are from the higher scores on these measures indicating worse quality of life.

### Interpretability

In the correlations between the change in the IWQOL-Lite scores and those of the Laval Questionnaire, the Pearson's coefficients were all > 0.5 (Table 5). This permitted our building of linear regression models in which the dependent variable was the difference in the Laval Questionnaire's scores, and the independent variable was the difference in scores on the IWQOL-Lite. The results are presented in Table 6. The best estimate of the MCID varied across domains and was in the range of 0.6 to 2.0 (always on a 7-point scale).

**Table 6:** Results of regression models using changes in the IWQOL-Lite to predict changes in the Laval Questionnaire

Laval Questionnaire's domains	Regression equation	Correlation coefficient (r)	D Laval Questionnaire corresponding to DIWQOL-Lite of 7.7*	D Laval Questionnaire corresponding to DIWQOL-Lite of 12.0*
D Symptoms	0.034 × IWQOL-Lite _Physical function _+ 0.38	0.73	0.64	0.78

D Activity/Mobility	0.055 × IWQOL-Lite _Physical function _+ 0.27	0.87	0.69	0.93

D Personal hygiene/Clothing	0.048 × IWQOL-Lite _Physical function _+ 0.84	0.74	1.21	1.42

D Emotions	0.035 × IWQOL-Lite _Self-esteem _+ 0.73	0.72	1.00	1.15

D Social interactions	0.044 × IWQOL-Lite _Public distress _+ 0.64	0.74	0.97	1.16

D Sexual life	0.024 × IWQOL-Lite _Sexual Life _+ 1.72	0.53	1.91	2.01

* MCID of the IWQOL-Lite = 7.7 to 12 on a 100-point scale; from reference [29].

## Discussion

This validation study indicated that the Laval Questionnaire represents a valid measure of health-related quality of life in patients with morbid obesity. It is sensitive to treatment-induced change, an essential property for its use in clinical trials.

We constructed the Laval Questionnaire from a study in which patients were asked to identify what they felt constituted the most significant items in their quality of life and to grade their importance [19]. This method ensured face and content validity of the new instrument. We used the "impact method" (instead of factor analysis) for item reduction and our clinical judgment for item clustering [19]. Although both methods may lead to the selection of different items, significant overlap usually exists when they are compared. Neither of the methods has proved superior to the other in selecting items to describe quality of life in specific health conditions [31]. The "clinical impact method" was selected for clarity and simplicity, and to preserve face and content validity. The only definitive way of deciding on the optimal approach would be to test the measurement properties of the instruments developed using the two strategies.

In the construct validity analyses, the high correlations between our questionnaire and the other related measures meeting our *a priori *predictions reinforce its validity [30]. However, most correlations with BMI (the only anthropometric measure included in our analysis) were only weak and not significant. A first explanation is that our patients represent a homogeneous population of patients with morbid obesity. Since all the spectrum of obesity was not represented in the population studied, this may have prevented our finding of obesity severity as a predictor of impaired quality of life. Another and widely accepted explanation is that, although a relationship between the level of BMI and quality of life impairment has been noted [4,5], this association is weak [6,7]. Also, we interpret the lack of correlation between the SES and the "emotions" domain of the Laval Questionnaire as an indication that both questionnaire measure different constructs, rather than poor validity of either of the questionnaires.

The Laval Questionnaire proved sensitive to change in quality of life in several ways. Statistically significant differences were observed in the patients who were submitted to bariatric surgery (Table 3). Also, large changes in scores we observed in treated patients, while small changes over time were seen in the control group (Table 4). We preferred the standardized response mean to assess an instrument's responsiveness for several reasons. It represents an intuitive estimate of the "signal-to-noise ratio" defining responsiveness [30]. In addition, it has direct implications for sample size determination for those planning clinical trials. The larger the standardized response mean, the smaller the sample size needed to demonstrate a treatment effect.

Perhaps the most important measurement property of a quality-of-life questionnaire used in clinical trials is its ability to reveal a minimal clinically significant change in a particular context. This property is referred to as "interpretability" that often relies on the determination of the MCID. Several methods have been described to determine the MCID. The "distribution-based methods" derive from measures of the score distribution of the instrument being explored [32]. Non-linearity of questionnaires undermines the legitimacy of this method. Also, these methods usually depend on the properties of the study sample. "Anchor-based methods" compare the changes in a studied instrument to other changes from other instruments. Anchor-based methods require an independent measure that is valid, that can be interpreted in itself, and that correlates, at least moderately, with the instrument being explored [33]. The method we used falls in the latter category. A limitation of our analysis comes from the fact MCID of the anchor we selected (i.e., the IWQOL-Lite) is only available for its total score, and not for its individual domains. Since we built linear regression models in which the independent variables were the differences in scores in individual domains of the IWQOL-Lite, we could provide only estimates of what may constitute the MCIDs of the Laval Questionnaire's domains. However, the determination of the MCID should be grounded in the experience of patients, not in statistics [33]. Only time and repeated utilization of the Laval Questionnaire will improve our understanding of its MCID.

Our study may also be considered as an independent validation study of the IWQOL-Lite that was developed and initially validated in a population of patients with obesity that cannot be qualified as "morbid" (mean BMI: 36.6 km/m^2 ^for women and 37.2 km/m^2 ^for men) [21]. We found that both the IWQOL-Lite and the Laval Questionnaire are valid and sensitive to change. Further validation is however necessary since our study was conducted in a single institution in patients who underwent biliopancreatic diversion with duodenal switch that represents 18% of the bariatric surgeries reported in a meta-analysis of clinical trials [34].

Generic questionnaire (such as the SF-36) have also been extensively used in obesity research. The SF-36 is actually the most utilized and recommended questionnaire to evaluate quality of life in obesity [18,35]. Although generic questionnaires are designed to measure all important aspects of quality of life, they are less likely to detect change in quality of life than disease-specific questionnaires which focus on specific areas of quality of life. As a consequence, generic questionnaires are usually less sensitive to change than disease-specific instruments, a situation that we also observed in our validation study (Table 3). We would suggest that future research includes further validation and a better definition of the interpretability of existing instruments, including ours.

## Conclusion

We conclude that the Laval Questionnaire is a valid measure of health-related quality of life in patients with morbid obesity and is sensitive to treatment-induced changes. The questionnaire is available on request. We believe that the Laval Questionnaire will be a useful tool in research and for clinical use. Further utilization of the questionnaire will determined the differences in score that may be regarded as the "minimal clinically important difference".

## Competing interests

The authors declare that they have no competing interests.

## Authors' contributions

All the authors contributed substantially to the conception and design of the protocol and to the acquisition, analysis and interpretation of the data. They also collaborated in drafting and revising the article critically for important intellectual content.

Specifically, FT managed the study from conception to publication. PM participated in the conception and design of the study and the revision of the manuscript. NT and SB carried out the patients' enrolment and the data collection. DR conceived and designed the study. YL managed the study from conception to publication.

All authors read and approved the final manuscript.
